# Parental care protects traumatized Sri Lankan children from internalizing behavior problems

**DOI:** 10.1186/s12888-015-0583-x

**Published:** 2015-08-25

**Authors:** Vathsalan Sriskandarajah, Frank Neuner, Claudia Catani

**Affiliations:** Department of Psychology, Bielefeld University, PO Box 100131, , D-33501 Bielefeld, Germany

## Abstract

**Background:**

Research in war-torn regions has mainly focused on the impact of traumatic experiences on individual mental health and has found high prevalence rates of psychiatric disorders in affected adults and children. However, little is known about the possible protective factors occurring in children’s environments in the aftermath of mass trauma. Therefore, we conducted a cross-sectional study with families in Northern Sri Lanka, a region that had been shattered by a long-lasting civil war and devastated by the Asian tsunami in 2004.

**Methods:**

Schoolchildren aged 7 to 11 (*N* = 359) were interviewed on the basis of standardized measures to assess children's exposure to traumatic events, mental health symptoms, and parenting behavior as perceived by children. All interviews were carried out by local senior counselors.

**Results:**

Linear regression analyses identified exposure to mass trauma and family violence as significant risk factors of child mental health whereas parental care emerged as a significant factor associated with fewer behavior problems. In addition, parental care significantly moderated the relationship between mass trauma and internalizing behavior problems.

**Conclusions:**

Family characteristics seem to be strongly associated with children’s mental health even in regions severely affected by mass trauma. This finding is particularly relevant for the development of targeted psychosocial interventions for children and families living in war torn areas.

## Background

Every year, thousands of people worldwide are affected by mass trauma, including war and other forms of armed conflicts [1], and also to a lesser extent, natural disasters such as floods, hurricanes and Tsunamis [2]. Children growing up in such adverse life environments are at risk of developmental problems [3, 4] and impaired psychological health and well-being [5, 6]. After the Asian Tsunami in 2004, studies with affected children in Thailand and in Sri Lanka reported high prevalence rates of posttraumatic stress disorder, major depression, and suicidal ideation [7–10]. High levels of internalizing and externalizing behavior problems were also found in children exposed to other natural disasters, such as Hurricane Katrina [11]. Across diverse countries and disaster types, the severity and frequency of exposure to disaster-related traumatic events was found to be one of the main predictors of psychopathology [12]. The linear relationship between disaster exposure and trauma-related disorders, which had been identified in previous studies with children [7] as well as with adults [13] has been referred to as dose-effect.

However, recent observations indicate that the assumption of a simple causal association between mass disaster and trauma-related impairment may be over-simplified. Beyond the individual level of analysis, and following Bronfenbrenner’s [14] bioecological system theory, it is likely that mass trauma does not only affect isolated individuals but also communities and families. Impairments in family and community functioning may, in turn, impair child adaptation and development [15] through secondary adversities such as parental loss, poverty, homelessness, community, and family violence [16]. Some recent findings support such a perspective. For example, family violence is increased in war or post-war communities [17–19] and associated with mass-trauma events [20]. Likewise, surveys conducted in the war-affected north of Sri Lanka showed that, in this region, an unusually high proportion of children had been affected by family violence. At the same time, family violence was more strongly associated with posttraumatic stress disorder than war exposure [7, 21] and predicted poor child adaptation measured by school performance, as well as mental and physical health [22]. These findings indicate that it is important to consider the multiplicity of adversities of children growing up in mass trauma regions as moderators of impairment [23].

However, even such an extended dose-effect perspective that includes secondary adversities as moderators neglects potential protective factors that have been emphasized in a risk-and-resilience perspective [24–26]. Protective factors or assets can be individual characteristics of the child’s developing biosystem but can also be found on the family as well as on the community level or in the societal and cultural context [4, 26]. Inter-individual different responses to mass trauma may result from individual factors such as gender, age, and developmental stage [4, 27]. Specific aspects of genetics and epigenetics may also promote resilience to trauma [28, 29]. On the community and family level, cultural beliefs and practices in mental health, resources in the family such as socioeconomic status, and the mental health of caregiver seem to function as protective factors of child’s mental health [30–32]. In a study with Chechen adolescents family support and perceived connectedness with family members were associated with lower levels of internalizing and externalizing behavior problems [33]. Given the high impact of the parent-child relationship and attachment for child development [34] the parenting behavior might not only act as additional stressor but also foster children’s resilience in the context of trauma and disaster [26, 35]. In this context, we regard the two-dimensional theory of the parent-child relationship as proposed by Parker and colleagues [36] as a useful framework for the identification of a potential protective factor. In line with the results of repeated clinical observations [37] and factor analytic studies of parental behavior [38], Parker and colleagues have identified *care* and *overprotection* as main characteristics that adequately describe a parent-child relationship. Based on these assumptions, we understand *parental overprotection* as authoritarian and intrusive parental behavior and *parental care*, also in accordance with the attachment theory [37, 39], as parental behavior that is characterized by emotional warmth and closeness. We operationalize parental care by using a measure of intimate bond developed by Parker and colleagues [36] where high parental care is equated with parental expression of affection, emotional support, and fair treatment and operationalized by specific parental behavior such as speaking to the child in a warm friendly voice, smiling frequently at the child, and understanding the child’s worries and problems. Similarly, low parental care is defined as parental behavior characterized by coldness, neglect, and rejection and operationalized by talking less to the child, ignoring the child’s needs, and making the child feel that he/ she is not wanted. In prior studies, low parental care as well as high levels of authoritarian and intrusive parenting were associated with poor psychological adjustment [40]. Furthermore, lack of parental care was identified as one of the strongest predictors for internalizing behavior problems in children (e.g. [41]). In line with these findings, studies with children and adolescents affected by natural and man-made disasters have even linked positive parenting behavior such as high parental care to children’s and adolescents’ resilience [26, 42, 43]. Children who had been exposed to the Hurricane Katrina reported fewer posttraumatic stress symptoms if their parents abstained from negative parenting behavior such as corporal punishment [44]. Likewise, adolescents exposed to the Tsunami reported fewer mental health symptoms if they had a highly caring mother [10]. Betancourt and colleagues [45] examined the psychological adjustment of former child soldiers from Sierra Leone and found that mental well-being was significantly correlated with perceived acceptance, understanding, and respect from family members.

However, the proof of a protective effect of parental care against the toxic effect of war adversities requires the documentation of a significant interaction between the experience of war trauma and parental care. Following this line of thinking, it is plausible to assume that parental care may not only be associated with better adjustment but might act as a protective factor for children’s mental health in the face of major adversities. In support of this, a limited number of studies indicate that supportive and positive parent-child relationship may have a moderating effect on children’s adjustment to mass trauma, both regarding symptoms of specific psychiatric diagnoses such as depression [10] as well as with respect to aggressive behavior [46]. Similarly, Punamaki and colleagues [47] found that war-affected Palestinian children who perceived only their mothers as highly loving and caring, but not their fathers, reported higher levels of posttraumatic stress symptoms compared to children who perceived both parents as loving and caring. However, so far research on the potential protective effect of parental care in the context of mass trauma has not focused on broader aspects of child mental health, such as internalizing and externalizing behavior problems.

Therefore, we conducted the present study with Sri Lankan school children living in a region with an almost unparalleled exposure to mass disaster including war and Tsunami. Sri Lanka’s civil war lasted over twenty-five years and came to an end only recently in May 2009 [48], 27 months prior to this survey. The Liberation Tigers of Tamil Eelam (LTTE) was fighting the Sri Lankan government aiming at political independence of the North-Eastern provinces of Sri Lanka [49]. According to independent estimates, up to 100,000 people were killed and approximately 800,000 people were displaced during the war [50, 51]. The final stage of the war took place in the Vanni area in spring 2009 and was characterized by an extraordinarily high level of violence with estimated 40,000 civil causalities [49]. Schools, hospitals, and roads were destroyed resulting in deprivation of water, food, and medical care in these areas. This war was fought while many parts of Sri Lanka were still recovering from the impact of the Tsunami catastrophe in 2004 that caused the death of more than 35,000 people and the forced displacement of another half million [52]. In this study, we included children from locations with varying exposure to war and Tsunami, to allow a high variance of both types of adversities in this sample. The present study aimed to explore the potential protective effect of parental care in the context of mass trauma. We predicted that mass trauma and family violence represent risk factors, whereas parental care serves as a protective factor for internalizing as well as externalizing behavior problems in children. Moreover, we assumed a moderating effect of parental care on the relation between mental health and mass trauma.

## Methods

### Sample selection

To investigate the effects of organized violence and natural disaster on family mental health and family relations we conducted a cross-sectional epidemiological survey with primary school children and their guardians in Jaffna, a district in the northern province of Sri Lanka, during the year of 2011. Overall, 359 children, 122 female guardians, and 88 male guardians were interviewed. The present paper focuses exclusively on the results based on the children’s sample.

The Jaffna district in Northern Sri Lanka was affected by the civil war as well as by the Tsunami in December 2004. Within this region, we purposely selected three clusters with varying levels of exposure to the civil war and to the Tsunami: *Valikamam West*, which is located near Jaffna downtown and was not affected by the recent war or by the Tsunami; *Valikamam North*, which was not affected by the Tsunami but was affected by earlier stages of the war, and *Vadamaradchi*, which was severely affected by both the Tsunami as well as the civil war. In each cluster two schools were selected and invited to participate. In each school, 60 children from 3^rd^ to 6^th^ grade were randomly selected from alphabetically-ordered lists of school children. In two schools, the total numbers of children from these grades were 63 and 62, so that all these children were included in the survey. None of the invited children refused to take part in the study. However, six children did not attend the interviews, because they were on vacation. Due to our limited time frame we could not re-invite them after their vacation. The implementation of the survey was approved by the Ethical Review Board of the German Society of Psychology (DGPs) as well as by regional school authorities in Northern Sri Lanka who had been the most reliable source of unbiased ethical review of research with the Tamil population in the immediate aftermath of the war.

### Participants

Altogether, 359 children took part in this study. Every other child was male (50.7 %) and on average they were 9.24 years old (SD = 0.98). Most of their fathers (95.5 %) and mothers (98.0 %) were alive, but 13.4 % of the children reported the loss of a sibling. The majority of the children were Hindus (59.9 % vs. Christian: 40.1 %). Almost half of the sample (46.9 %) reported at least one displacement due to civil war, Tsunami or flood. The number of rooms in a household ranged from 1 to 10 (M = 2.66; SD = 1.15) and the number of persons (including reporting child) from 2 to 13 (M = 6.18; SD = 1.63). On average, the children consumed 3.36 meals and snacks per day (SD = 0.79) and possessed 14 pieces of clothes and toys (SD = 9.16).

### Measures

We mainly used instruments that had previously been translated into Tamil language and validated. Additional questionnaires were translated following international standards [53, 54]. Initially the instruments were translated from English to Tamil language by local counselors. Tamil versions were then translated back into English by translators, who were blind to the original version. Subsequently, local experts reviewed disagreements between the original and the translated items and discussed them with a team of translators and local counselors to optimize conceptual and semantic equivalence as well as acceptability and relevance to the local culture. All measures, including those originally designed as self-report questionnaires, were used in interview form with the children.

#### Sociodemographic characteristics

The sociodemographic questionnaire and the assessment of the Tsunami exposure were adopted from previous studies in Sri Lanka [7, 55].

#### Exposure to traumatic and stressful life events

Family violence experienced by children was defined as exposure to physical, emotional or sexual abuse as well as witnessing intimate partner violence between parents. For the assessment of adverse childhood experiences at home we used a questionnaire which had previously been used with children in the Tamil context [7]. The amount of war-related exposure was established by counting the different event types that were reported by the child separately for war-related events that happened during the 12 months preceding the interview (scored as “last year”) and those that happened more than 12 months prior to the interview (scored as “ever”). The family violence questions aimed at determining whether events had happened “last month” or “ever”. Thus, it was possible to determine the number of cases in which there was ongoing family violence. In addition, a simplified version of the Life Events Checklist (LEC) was administered in order to assess other potentially traumatic events besides war, Tsunami, and family violence [56]. The subjects were only asked whether they had experienced any of the events or witnessed them. The question whether they had learned about any event happening to someone else close to them was omitted.

#### *Posttraumatic Stress Disorder*

To assess Posttraumatic Stress Disorder (PTSD) the University of California of Los Angeles (UCLA) PTSD Reaction Index for DSM-IV (UPID) for children was used [57]. This instrument has been used in a variety of cultural settings and has shown overall good validity and nearly excellent reliability [58]. In a previous study, the UPID had been translated into Tamil following standard principles of instrument translation and validated [55]. Rather than relying on a cut-off criterion, we established the diagnosis of PTSD according to the fulfillment of the DSM-IV criteria assessed through the corresponding items. For this purpose, we added six items related to problems in functioning in different areas of children’s life [7].

#### Depression, dhysthymia and suicide

Sections A, B, and C of the Mini-International Neuropsychiatric Interview for Children and Adolescents (MINI-KID; [59]) were used for the assessment of major depression, dysthymia, and suicidality. The MINI-KID is a structured clinical interview designed to assess psychiatric disorders in children and adolescents according to DSM-IV (Diagnostic and Statistical Manual of Mental Disorders, Fourth Edition) and ICD-10 (International Classification of Diseases, 10th Revision). It has shown good reliability and good concurrent validity with the parents’ version of the MINI-KID (MINI-KID-P) and the Schedule for Affective Disorders and Schizophrenia for School Aged Children – Present and Lifetime Version (K-SADS-PL; [60]).

#### Emotional and behavioral problems

Psychosocial adjustment was assessed by use of the Strengths and Difficulties Questionnaire (SDQ) self report version [61]. The SDQ is subdivided into five scales with five items each: emotional symptoms, conduct problems, hyperactivity/inattention, peer relationship problems, and prosocial behavior. Items from the emotional symptom scale and the peer relationship problem scale can be combined into an “internalizing behavior” subscale (range: 0–20) and the conduct problems and hyperactivity items into an “externalizing behavior” subscale (range: 0–20). The total difficulties score (range: 0–40) is determined by aggregating the indices for internalizing and externalizing behavior problems. The SDQ can be completed by children and adolescents (self-report) or caretakers or/ and teachers. This questionnaire has been used in different countries and has consistently shown good psychometric properties [62]. A translated and validated Tamil self-report version was used in this study [63].

#### Parental care and overprotection

Two core dimensions of parenting, care and overprotection, were measured according to the child’s responses for each parent on the Parental Bonding Instrument (PBI; [36]). The PBI consists of 12 items about care and 12 items about overprotection. The two scales are commonly used to determine four different types of parenting, (1) optimal bonding (high care and low overprotection), (2) affectionate constraint (high care and high overprotection), (3) affectionless overprotection (low care and high overprotection), and (4) neglectful parenting (low care and low overprotection). The PBI has shown good reliability and validity across different cultural settings (e.g. [64]). Although the questionnaire had originally been designed for adults as a retrospective tool, it has also been employed in several studies with teenage adolescents (e.g. [65]). Furthermore, a comparison between mother interviews and child reported PBI scores supports the validity of the PBI as a measure of not only perceived but also observed parental characteristics [66].

### Procedure

Nine former schoolteachers, who had been extensively trained as “Master Counselors” and had also engaged in a number of previous studies of our workgroup [8, 67], conducted the clinical interviews with children. We relied on the use of clinical interviews in order to minimize the rate of missing data, misunderstandings, and reporting bias. In a 10-day workshop, the Master Counselors were provided with detailed instructions on conducting the study with the children. Frequent supervision meetings with one clinical psychologist from our work group and peer consulting groups were scheduled to ensure that the local team was properly supported and clinical interviews were carried out correctly. The parents were invited to their children’s school and informed about the survey. The interviews were based on the questionnaires outlined above and took place on the school grounds. Prior to the interview, written informed consent was obtained from the children and one of their parents or legal guardians.

### Data analysis

Data were analyzed using the Statistical Package for Social Sciences (SPSS), Windows Version 20 (Chicago, Illinois, USA), and JMP 6.0 (SAS Institute). To describe the traumatic events experienced by the children, their mental health and the perceived parenting style we calculated frequencies, mean scores, and standard deviations. Differences between groups were examined using the Kruskal-Wallis test. Spearman’s rank correlations (Spearman’s ρ) were used to assess the bivariate association between maternal and paternal care and overprotection. In order to determine the risk and protective factors associated with children’s mental health we conducted linear regressions analyses with hierarchical linear models. In all models, school and location were entered as nested fixed factors to adjust for potential cluster effects. Rather than relying on specific psychiatric diagnoses we used the scores of the externalizing and internalizing symptoms as outcome measures to identify predictors of broader aspects of child mental health, such as emotional and behavioral problems. Sex, age, socioeconomic status (SES), number of family violence related events, number of mass trauma, and parental care were entered as potential predictors. The interaction between the amount of experienced mass trauma and parental care was added as a further variable in the regression model. This interaction was further analysed using Scheffe’s post-hoc tests for multiple comparisons.

## Results

### Exposure to adverse and traumatic events

#### War exposure

Every sixth child (16.4 %) experienced at least one type of war related event in the past year. More than one third of the sample (38.4 %) reported the experience of at least one war event during their lifetimes. The children reported on average 1.2 (SD = 2.0) different types of war events in their life. As intended by the sampling procedure, reports of war exposure depended on locations, with higher values from more affected regions. Across all regions, seeing a dead or mutilated body (23.7 %), being close to shelling or gunfire (21.4 %) and being rounded up (17.5 %) were the war related events most often mentioned by the children.

#### Family violence

**Fig. 1:**
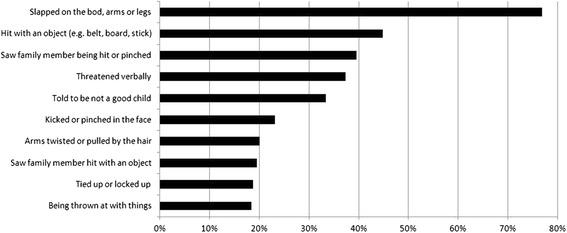
Frequency (%) of family violence event types reported most often

The majority of the sample (85.5 %) reported at least one family violence related event. On average, the children experienced or witnessed 4.5 (SD = 4.2) different event types in their families. Figure 1 shows the frequency of the ten family violence related events most often reported by the children. At least one injury as a result of the reported incidents was mentioned by 12.8 % of the children and 5.0 % needed medical treatment because of the experienced violence. Sexual abuse at home was experienced by 3.1 % of the children. Almost half of the sample (45.4 %) witnessed at least one incidence of violence in their families. Most of the children (73.8 %) stated that the violence at home was ongoing.

#### Traumatic life events

The most frequent traumatic event types reported by the children were experiencing (54.5 %) and witnessing (47.4 %) a physical assault. Almost one third (29.9 %) of the children stated, that they experienced a natural disaster. Apart from the Tsunami in 2004 the experience of the Cyclone Nisha in 2008 was the only other natural disaster mentioned by the children. As expected, children in the coastal area were found to be considerably more affected by the Tsunami (46.1 %) than children in the other two regions (0.8 % respectively).

### Psychological problems and mental disorders

#### Traumatic stress and PTSD

Every other child (49.9 %) reported an event that fulfilled the A criterion for the PTSD diagnosis based on DSM-IV. Living in a combat or war zone was rated most frequently by the children as their most upsetting life event (12.2 %) followed by experiencing (8.5 %) and witnessing (9.1 %) physical assault. Natural disaster was reported by 7.3 % as their most upsetting life event. In line with the variable degree of exposure to mass trauma in the three different areas where children were recruited, PTSD prevalence varied in accordance with the location, with a prevalence rate of 1.7 % in Valikamam West, 5.6 % in Valikamam North, and 33.6 % in Vadamaradchi.

#### Major depression, dysthymia, and suicidality

The criteria for a diagnosis of major depressive disorder according to the DSM-IV was fulfilled by 3.9 % of the children. The prevalence of Major Depression ranged from 0 % in Valikamam West to 7.0 % in Vadamaradchi. 5.7 % of the children (range: 0–17.3 %) were diagnosed with a dysthymic disorder. 11.2 % of the children (range: 0–25 %) had suicidal tendencies in the past and 9.2 % (range: 0–23.3 %) were diagnosed with current suicidal ideation according to the Mini International Neuropsychiatric Interview (MINI).

#### Internalizing and externalizing behavior problems

**Fig. 2:**
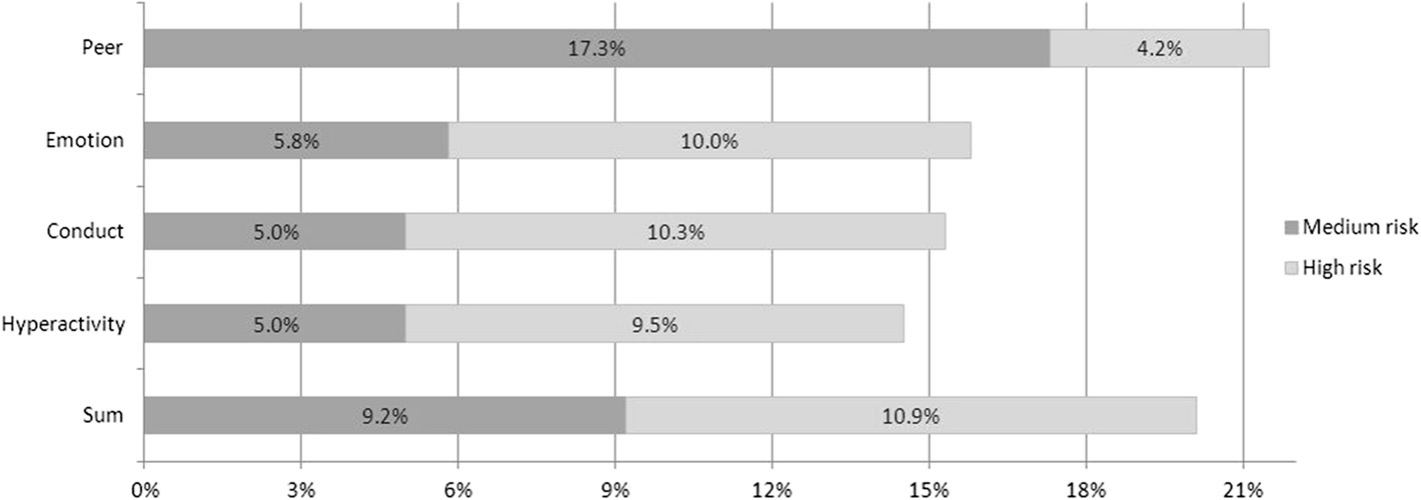
Frequencies (%) of critical scores on the five SDQ subscales

The mean for internalizing problems was 4.7 (SD = 3.7; obtained range: 0–16) and the mean for externalizing behavior problems 5.1 (SD = 3.5; obtained range: 0–17). The average total score of the SDQ was 9.8 (SD = 6.7; obtained range: 0–32). The percentages of children with medium and high risk for mental disorders are shown in Fig. 2.

### Perceived parenting behavior

The mean for paternal care as perceived by the children was 28.5 (SD = 5.8) whereas the mean for maternal care was 29.4 (SD = 5.5). The means for parental overprotection were 16.3 for fathers and 16.5 for mothers (both SD = 3.5). There was a significant correlation between paternal and maternal care (Spearmans’ ρ = .78, *p* < .001) as well as between maternal and paternal overprotection (Spearmans’ ρ = .66, *p* < .001). According to the cut-offs established by the authors of the PBI [36], perceived parenting styles were assigned to the four different quadrants. These cut-offs have also been successfully employed in Asian settings before [68]. Table 1 shows the distribution of the proportion of Tamil children in each quadrant referring to perceived maternal and paternal care and overprotection.

**Table 1:** Perceived parenting style of parents or primary caretakers

	Mother or primary female caretaker (*N* = 359)	Father or primary male caretaker (*N* = 336)
Affectionate constraint (high care & high overprotection)	49.9 % (*N* = 179)	64.0 % (*N* = 215)
Affectionless control (low care & high overprotection)	29.5 % (*N* = 106)	21.7 % (*N* = 73)
Optimal parenting (high care & low overprotection)	13.9 % (*N* = 50)	10.4 % (*N* = 35)
Neglectful parenting (low care & low overprotection)	0.8 % (*N* = 3)	0.9 % (*N* = 3)

### Prediction of child mental health

In order to identify the risk and protective factors of mental health we conducted two linear regressions analyses with the internalizing behavior problem score and the externalizing behavior problem score of the SDQ as dependent variables. In addition to age and gender, socioeconomic status was entered as a predictor. An index of socioeconomic status (SES) was computed as the mean of the following standardized (z-transformed) variables: Number of meals and snacks per day, number of toys, number of clothes, possession of a bank account, possession of an own bed/mat, and ratio of the number of rooms to the number of persons in a household. The mean of perceived paternal and maternal care was aggregated together to one index of parental care. In 23 cases where the scores related to fathers were missing, because the father had died (*N* = 16), was living separated from the family (*N* = 6) or was missing (*N* = 1), we used the maternal care score only. Based on a median-split we categorized the scores in “low” and “high parental care”. The number of family violence events and the number of mass trauma were entered as predictors representing adverse life events. Exposure to mass trauma was grouped into three categories of increasing severity (amount): (1) no exposure to mass trauma, (2) exposure to one type of mass trauma (Tsunami OR war), and (3) exposure to war AND Tsunami. Finally, we computed the interaction term “number of experienced mass trauma x parental care” following the lead of Caspi and colleagues [69] and entered it as a further independent variable. Nine children were excluded due to missing data in the socioeconomic questionnaire or the family violence event check list. The results of the regression analyses in Table 2 show that the experience of violence at home was the strongest predictor of internalizing and externalizing behavior problems. Apart from exposure to mass trauma, parental care was the most significant predictor of externalizing behavior problems in children and was an even stronger predictor of internalizing behavior problems in children. The interaction term of exposure to mass trauma and parental care was significant only for internalizing behavior problems. The residuals in both regressions were normally distributed (*Kolmogorov-Smirnov-Z* > 1.17, *p* > .10).

**Table 2:** Predictors of internalizing and externalizing behavior problems and significance levels resulting from linear regression modelling

	Internalizing behavior problems		Externalizing behavior problems	
Predictor variable	Zero-order correlation	Standard ß-coefficent	Zero-order correlation	Standard ß-coefficent
Sex [male]	-.04	-.03	-.06	< −.01
Age	.18**	.07	.10	< .01
SES	.04	.03	-.01	-.03
Number of family violence related events	.55***	.39***	.64***	.44***
Number of mass trauma	.50***	.34***	.50***	.24***
Parental care [low]	.43***	.20***	.47***	.11*
Number of mass trauma x Parental care	.23***	.16**	.32***	.07

Internalized behavior problems: Adjusted *R* ^*2*^ = .42; *F*(9, 340) = 28.53; *p* < .001. Externalized behavior problems: Adjusted *R* ^*2*^ = .47; *F*(9, 340) = 35.42; *p* < .001. *p < .05. **p < .01. ***p < .001. Controlled for location (included as a fixed factor)

#### Interaction of exposure to mass trauma and perceived parental care

**Fig. 3:**
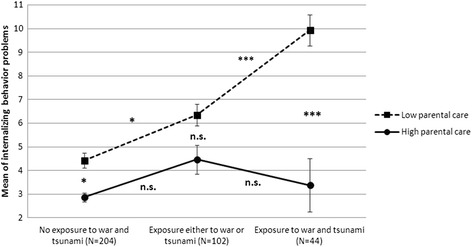
The interactive effect of children’s war and Tsunami exposure and perceived parental care on internalizing behavior problems in children (*N* = 350). Means, standard errors, and Scheffé’s test results are shown. *p < .05. ***p < .001

Figure 3 illustrates the moderating effect of parental care on the relation between exposure to mass trauma and internalizing behavior problems. Scheffé’s test results showed that in children who perceive their parents as highly caring, the experience of mass trauma is not associated with a greater level of internalizing problems as opposed to children in the “low care” group.

## Discussion

The present study investigated risk and protective factors for the mental health of children growing up in an adverse post-conflict environment facing the consequences of decades of civil war and a devastating natural disaster. Our main finding is that in a context of multiple trauma, parental care moderates the relation between the experience of mass trauma and children’s internalizing behavior problems. Thus, parental care seems to be a powerful protective factor that is able to mitigate the adverse effects of trauma due to war and natural disaster on children’s mental health. The present findings also reaffirm the harmful consequences of different adverse and traumatic experiences, such as exposure to war or family violence, for children’s mental health.

A considerably high number of children in the present sample was affected by mass trauma, i.e. war and natural disaster. More than one third of the children reported adverse experiences related to the Sri Lankan civil war, whereas up to 46 % of the children (those living in the coastal areas) had been exposed to the Tsunami. In agreement with previous findings in war-torn communities [7, 20, 21], family violence was an additional stressor for the Tamil children in our study, with nearly all of them having experienced violence within the family at least once. The finding of increased rates of family violence towards children in post-war populations corroborates the notion that mass trauma typically leads to secondary adversities on various levels, for instance family disruption and loss, homelessness or impaired access to education and health services [15, 16]. Frequent incidences of family violence and the use of negative child rearing strategies can be seen as further examples of such secondary adverse factors. In the present sample, a considerable percentage of children reported high parental care as well as high parental overprotection indicating a parenting style of “affectionate constraint”. Similar parenting types had been also reported by adolescents from India [70]. The co-occurrence of high parental care and high parental overprotection in these populations may result from a common Asian assumption that control and overprotection are both equally relevant aspects of parental attention and care. As a consequence, parental strictness and overprotection are not necessarily perceived as negative child rearing strategies [71]. It is also conceivable that parents may become more overprotective of their children in the context of living with community-wide disasters that cause the loss of lives and property. For example, evidence shows that the loss of a child may have an impact on parents’ behavior towards the remaining children [72].

In line with previous findings in war affected communities [7, 53], the high trauma load in Tamil children was reflected in their mental health outcomes, in particular in a PTSD prevalence rate of up to 33.6 % as well as rather high percentages of Major Depression and suicidal tendencies. Interestingly, the rates of internalizing behavior problems were much higher than the rates of externalizing behavior problems. A reason for such a result may be the impact of culture and society: the Sri Lankan society is mainly coined by collectivist ideas and thus conformity and high functioning is expected of the individual by society [73]. Externalizing behaviour problems may therefore be less acceptable. In fact, studies comparing British children with children from India revealed higher rates of internalizing behavior problems in Indian children [74].

Consistent with findings from previous studies [7, 21, 22] both exposure to mass trauma (war and Tsunami) as well as experiences of family violence were independently associated with mental health problems in children, with regard to internalizing as well as externalizing behavior problems. This result is in line with earlier studies showing a dose-effect of traumatic stress on children’s psychosocial functioning [7, 23] whereby exposure to an increased amount of stressors is related to a higher prevalence of mental health disorders or poorer adaptation.

However, the present data also indicate that a dose-effect assumption is an insufficient explanation of the effects of traumatic events on childrens mental health. Instead, the adoption of a risk-and resilience perspective on the effects of extreme adversity on children [24, 25] which strengthens the relevance of protective factors, seems to be appropriate in this context. Results of the regression analysis revealed parental care as a significant factor which contributes to better mental health in children regarding both, externalizing and particularly internalizing behavior problems. More specifically, we found that parental care significantly moderates the association between exposure to mass trauma and internalizing behavior problems in children. The present data indicate that the impact of parental care may be so decisive that it can annihilate the dose effect of mass trauma on children’s psychological wellbeing. Those children who reported their parents to be highly caring did not show significant increases in internalizing behavior problems related to the exposure to mass trauma. This finding is consistent with the general assumption that parent-child relationship may be one of the core variables influencing children’s psychological adaptation even in the aftermath of mass trauma [26] and the suggestion of Gewirtz and colleagues [35] that parenting practices may regulate children’s adjustment in the aftermath of trauma exposure. They support earlier findings in other war-affected child populations that supportive and positive parent-child relationship can have a moderating effect of children psychopathological reactions to traumatic experiences [10, 46]. However, research on parenting and child care and their relationship with child adaptation in communities affected by mass trauma is still rather scarce.

This interaction effect was restricted to internalizing behavior problems, whereas Qouta and colleagues [46] also found a moderating effect on aggressive behavior. The protective role of parental care for internalizing behavior problems is not surprising as it has also been suggested by longitudinal studies on parenting styles and mental health which have shown that lack of parental care is associated particularly with symptoms of depression (e.g. [75]). There is an assumption that unpleasant interactions with the parents lead to emotional distress in children, and that children who experience their parents as less caring may develop negative cognitions about themselves and the world [76].

Some limitations of the present study are worth noting. Due to logistical and political restrictions during the time of the survey it was not possible to randomly select the regions and schools, so that our sample is not representative for the whole community of Sri Lankan Tamils. Still, the available sample appeared to be very similar to the community of school children in the Jaffna region with respect to traumatic war and Tsunami exposure, displacement history, and socioeconomic status. The current study focused on specific aspects of child mental health, namely PTSD and depression, as well as emotional and behavioral problems measured by the SDQ. Future studies with children in post-conflict settings should include more extensive diagnostic interviews that allow for the diagnosis of a broader range of psychological disorders. Most importantly, due to the cross-sectional design of our study, it is premature to consider the protective effect of parenting as proven, especially since the parenting experiences were assessed in an interview using self-report measures. Although several studies indicated that rating of parents’ behavior is stable even over a 20 year period [77] a reporting bias may have contributed to our results. Future studies should add behavioral observations as independent measures of parenting behavior and study the temporal course of parenting and mental health in longitudinal studies.

## Conclusions

Our results strengthen the view that parenting practices including familial violence play a major role for the wellbeing of children living in post-conflict settings characterized by experiences of mass trauma (e.g. [17]). Parenting practices should not only be seen as potential risk factors for children’s positive development but also as a variable that can have a strong protective impact. In fact, high parental care and attention towards the child seem to alleviate the negative impact that mass trauma such as war and natural disasters have on children’s mental wellbeing even in a highly adverse context such as a post-conflict region. The present findings need to be replicated in larger and more representative samples. However, it is hoped that these findings may stimulate debate about adequate interventions for children affected by mass trauma and those who live in the aftermath of war and disaster. So far, psychological interventions have often focused on the individual by addressing trauma symptoms and related mental health problems in affected children [78, 79]. Given the decisive role of parental care for the adaptation of children towards mass trauma, intervention programs should include culturally sensitive family based approaches and parenting trainings that meet the specific needs of families and children in post-war communities. Even though a small number of family-based interventions have been developed and adapted for the use with post-war communities [80, 81], the empirical evidence regarding their long-term efficacy is still scarce. Future research should aim at filling this gap and further develop and test family-level interventions that may be effective and sustainable in promoting resilience in children in post-war societies.
